# Emotional intelligence leadership and career decision-making self-efficacy among college students in China: The mediating role of social support and proactive personality

**DOI:** 10.1371/journal.pone.0343432

**Published:** 2026-02-23

**Authors:** Kaida Wang, Jun Hu, Xiao Yang, Hua Ding, Hailun Huang, Zhanlu Xu

**Affiliations:** 1 School of Marxism, Zhejiang University, Hangzhou, China; 2 School of Economic, Hangzhou Normal University, Hangzhou, China; 3 School of Marxism, Hangzhou Normal University, Hangzhou, China; 4 School of Public Health, Hangzhou Normal University, Hangzhou, China; 5 School of Material Chemistry and Chemical Engineering, Hangzhou Normal University, Hangzhou, China; National University of Sciences and Technology, PAKISTAN

## Abstract

Student leadership education is a significant component of global education that positively impacts college students’ employment. This study examined the relationship between emotional intelligence leadership and career decision-making self-efficacy, specifically investigating the mediating roles of social support and proactive personality. A cross-sectional survey was conducted among 996 university students in China (314 males, 682 females, aged 18 to 23) using the Emotional Intelligence Leadership Scale, Career Decision-Making Self-Efficacy Scale, Social Support Scale, and Proactive Personality Scale. Structural equation modeling revealed that emotional intelligence leadership was significantly and positively correlated with career decision-making self-efficacy. Furthermore, social support and proactive personality mediated this relationship through three distinct pathways: independent mediation by social support, independent mediation by proactive personality, and a serial mediation involving both factors. These findings contribute to the understanding of how emotional intelligence leadership facilitates career development. Educational institutions are encouraged to enhance emotional intelligence leadership education and foster supportive social environments to bolster students’ career decision-making self-efficacy.

## 1. Introduction

In recent years, university graduates have faced increasingly severe employment challenges, particularly within the Chinese context [1,2]. Many studies have shown that career decision-making self-efficacy is a critical predictor of employment success [3–6]. While these studies often focus on general psychological factors and social support, the specific role of emotional intelligence leadership in fostering university students’ career decision-making self-efficacy remains systematically underexplored. The exact role, mechanisms, and evidence of emotional intelligence leadership in career decision processes remain unclear [7–10].

Leadership development has long been a focal point in global higher education [11–13]. Research demonstrates strong correlations between student leadership competencies and post-graduation employment performance, income levels, and related outcomes [1 4–17]. However, most existing research focuses on general leadership skills and pays little attention to the dimensions of emotional intelligence leadership that emphasize emotional regulation, empathy, and motivation [18]. Within Chinese higher education contexts, students’ career decisions are profoundly shaped by familial and social network influences [19–20]. Emotional intelligence leadership may be associated with students’ greater career decision confidence, an association that might be evident through factors such as positive affect, social support, and proactive behaviors. Nevertheless, research in this domain remains limited.

To address this gap, this study draws upon Social Cognitive Career Theory (SCCT) to investigate the relationship between emotional intelligence leadership and students’ career decision-making self-efficacy. We specifically explore the potential mediating roles of social support and proactive personality in this association. This research contributes by: (1) offering a clearer theoretical model of how emotional intelligence leadership functions in career decision-making, which expands on current self-efficacy research; and (2) practically providing more targeted intervention strategies for leadership education and career counseling in universities, potentially alleviating the intensifying employment challenges faced by graduates. Ultimately, we seek to elucidate how emotional intelligence leadership and career decision-making self-efficacy are associated, while establishing foundational insights for future cross-cultural studies and educational practices.

## 2. Literature review

### 2.1 Emotional intelligence leadership and career decision-making self-efficacy

Emotional intelligence leadership is a developing, process-oriented form of comprehensive leadership [21–23]. While its role in supporting organizational performance has been widely explored in the field of organizational behavior [24,25], research in higher education also indicates that it is closely associated with higher levels of career decision-making self-efficacy [26–28].

Career decision-making self-efficacy refers to an individual’s confidence in their ability to successfully complete tasks related to career decision-making [29,30] and serves as a critical predictor of job-seeking behavior [31]. Individuals possessing high emotional intelligence leadership are adept at utilizing emotional experiences to guide their thinking and actions during career planning [32,33]. By effectively managing self-efficacy expectations and reducing anxiety and fear related to career choices [34], they enhance their confidence in pursuing career tasks [35,36].

Based on this, we hypothesize that：H1: Emotional intelligence leadership has a significant positive impact on career decision-making self-efficacy.

### 2.2 The mediating role of social support

Social support refers to the assistance provided by family, friends, and social institutions to meet an individual’s various needs [37], with high levels of support contributing to enhanced psychological resilience [38]. Emotional intelligence leadership encompasses empathy and social skills, which facilitate the establishment of trust and cooperation [39,40], thereby promoting the formation and maintenance of social support networks [41–43]. Individuals with this trait demonstrate superior performance in team communication and conflict resolution [4,23], making them more adept at collaborative processes and acquiring social support [44,45]. Research indicates a significant positive correlation between social support and career decision-making self-efficacy [46–48]. According to the buffering model of social support, external support can alleviate psychological stress and enhance positive emotions [49–51]. In the context of higher education, improving emotional intelligence leadership helps students build supportive networks [52,53]; such support boosts individual confidence, subsequently elevating career decision-making self-efficacy [54].

Based on this, we hypothesize that：H2: Social support significantly mediates the relationship between emotional intelligence leadership and career decision-making self-efficacy.

### 2.3 The mediating role of proactive personality

Proactive personality is a stable trait characterized by an individual’s ability to identify and utilize opportunities to improve their circumstances, irrespective of situational constraints [55]. Studies show that emotional intelligence leadership is positively correlated with proactive personality: emotionally intelligent leaders cultivate a sense of agency and responsibility by fostering supportive atmospheres and effectively managing emotions [56–58]. This leadership style creates psychological safety, encouraging individuals to explore novel solutions and actively adapt to challenges rather than waiting passively [59]. In the domain of career development, proactive personality is a strong predictor of career decision-making outcomes [60] and is significantly positively associated with career decision-making self-efficacy among college students [2,61,62]. Highly proactive individuals tend to actively gather information and enhance skills, thereby building a base of mastery experiences and social persuasion, which are key sources for strengthening self-efficacy [63,64]. Taken together, emotional intelligence leadership activates individual proactivity, which subsequently enhances career decision-making self-efficacy through positive feedback from mastery experiences [65–67].

Based on this, we hypothesize that：H3: Proactive personality significantly mediates the relationship between emotional intelligence leadership and career decision-making self-efficacy.

### 2.4 The potential chain mediation effect

Based on Social Cognitive Career Theory (SCCT) [68], we propose a serial mediation model integrating the aforementioned pathways. While H2 and H3 address parallel mediation, SCCT suggests a dynamic interplay where environmental factors can shape personal attributes. We specifically posit a link between social support and proactive personality.

Research indicates that social support acts as a “secure base,” providing the emotional and informational resources necessary for individuals to engage in proactive behaviors [69,70]. When students perceive reliable external support, they feel greater psychological safety, which encourages them to take initiative, explore career options, and embrace challenges—hallmarks of a proactive personality [2,62].

Therefore, we hypothesize that EIL not only directly influences CDSE but also initiates a sequential process: EIL facilitates the acquisition of social support [41], which in turn fosters a proactive personality, ultimately enhancing career decision-making self-efficacy [71,72].

Therefore, we hypothesize that：H4: Social support and proactive personality jointly function as a chain mediating mechanism between emotional intelligence leadership and career decision-making self-efficacy.

### 2.5 Conceptual framework

**Fig 1:**
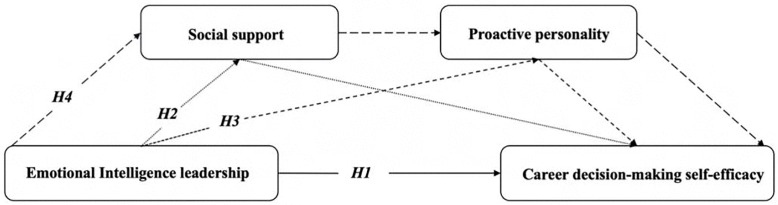
The hypothesized conceptual framework.

Based on the Social Cognitive Career Theory and the hypotheses proposed above, we developed a conceptual framework to illustrate the mechanism linking emotional intelligence leadership to career decision-making self-efficacy. As depicted in Fig 1, the model positions emotional intelligence leadership as the independent variable and career decision-making self-efficacy as the dependent variable. Social support and proactive personality are incorporated as mediators. The framework integrates four pathways: the direct effect of emotional intelligence leadership on self-efficacy (H1), and the indirect effects through the parallel mediation of social support (H2) and proactive personality (H3), as well as the serial mediation where social support enhances proactive personality (H4).

## 3. Methods

### 3.1 Participants

This study adopted a cross-sectional design, randomly selecting university students from ten regions in China through university student networks and social media platforms. Before completing the questionnaire, all participants were required to read and agree to an informed consent form, which outlined the study’s purpose, data confidentiality measures, voluntary participation rights, and survey completion details. Participants were explicitly informed that all responses would remain anonymous and used solely for academic research, and that they could withdraw at any time without any consequences. Only those who clicked the “I agree to participate” button were able to proceed with the survey.Initially, 1,100 students were recruited, and after excluding responses that were incomplete, completed in under 30 seconds, or contained identical answers to all items, a total of 996 valid responses were retained (314 males, 682 females, aged 18–23; see Table 1), yielding a response rate of 90.5%. Participants were from five academic years: first-year (51.3%), second-year (13.7%), third-year (24.1%), fourth-year (8.8%), and fifth-year (2.1%). The sample represented a diverse range of disciplines, with the most common fields being economics (25.6%), medicine (22.3%), agriculture (15.1%), literature (10.6%), and science (8.9%), along with participants from other academic backgrounds.

**Table 1:** Descriptive Statistics of Socio-Demographic Variables (N = 996).

		*Total N (Percentage)*
**Gender**	Male	314(31.5)
	Female	682(68.5)
**Age**	18	142(14.3)
	19	330(33.1)
	20	188(18.9)
	21	177(17.8)
	22	115(11.5)
	23	44(4.4)

### 3.2 Measures

#### 3.2.1 Emotional intelligence leadership scale.

The Emotional Intelligence Leadership scale used in this study was adapted from Tao revision of the Chinese Student Emotional Intelligence Leadership Scale, originally based on Shankman and Allen’s model [71,73]. The scale comprises three sub-scales: situational awareness, self-awareness and other-awareness. Each item assesses the frequency of specific leadership behaviors using a 5-point Likert scale ranging from 1 (never) to 5 (always). A higher total score indicates a higher level of emotional intelligence leadership, with 35–40 indicating high, 26–34 medium, and 8–25 low levels. This scale also demonstrated good internal consistency in this study (Cronbach’s α = 0.945) [74]. The Cronbach’s α coefficients for situational awareness, self-awareness, and other-awareness were 0.928, 0.920, and 0.932, respectively, indicating acceptable reliability. Confirmatory factor analysis (CFA) results indicated a good model fit: χ²/df = 4.152, CFI = 0.953, TLI = 0.948, RMSEA = 0.056 [75].

#### 3.2.2 Social support scale.

The Social Support Scale, developed by Xiao, was employed to assess social support [76]. The scale comprises three sub-scales: subjective support, objective support and support utilization. Scores above 40 reflect high social support, scores between 20 and 40 indicate medium social support, and scores below 20 represent low social support. Research has indicated that the Cronbach’s α for this scale is 0.9284. In this study, the scale demonstrated good internal consistency (Cronbach’s α = 0.782), with Cronbach’s α values of 0.928, 0.920, and 0.932 for subjective support, objective support, and support utilization, respectively, indicating strong reliability.

#### 3.2.3 Proactive personality scale.

The Proactive Personality Scale, translated and revised by Shang and Gan from Bateman and Crant’s original scale, was employed to assess proactive personality [77]. The scale comprises 11 items, rated on a 7-point Likert scale ranging from 1 (strongly disagree) to 7 (strongly agree). Higher scores reflect greater levels of proactive personality. Studies have reported that the overall Cronbach’s α for this scale is 0.8685, In this study, the scale demonstrated excellent internal consistency (Cronbach’s α = 0.950), with a single-factor total variance contribution rate of 66.710% and factor loadings ranging from 0.803 to 0.834, indicating strong construct validity.

#### 3.2.4 Career decision-making self-efficacy scale.

The Career Decision-Making Self-Efficacy Scale employed in this study is a simplified version of the scale developed by Betz and revised by Long [78,79]. The scale comprises five sub-scales: self-appraisal, occupational information gathering, goal selection, planning and problem-solving. Each item is rated on a 5-point Likert scale (1 = no confidence at all, 5 = complete confidence). Higher scores reflect greater career decision-making self-efficacy. One study found the Cronbach’s α of this scale to be 0.8953, and in the present study, it exhibited good internal consistency (Cronbach’s α = 0.958). The Cronbach’s α values for the sub-scales were 0.903, 0.879, 0.904, 0.906, and 0.896, respectively, indicating strong reliability. Additionally, CFA results indicated a good model fit: χ²/df = 2.992, CFI = 0.969, TLI = 0.965, RMSEA = 0.045.

### 3.3 Procedure

The questionnaire was administered through Wenjuanxing, a widely used online survey platform in China that provides secure data collection, customized reporting, and analytical tools. The survey was converted into a QR code and distributed via WeChat and DingTalk to ensure broad accessibility among university students, who could access and complete the survey at their convenience using mobile phones or computers. The questionnaire consisted of 78 questions, with a completion time of M = 605.57 seconds (SD = 170.94 seconds, Min = 300 seconds, Max = 1293 seconds, Median = 604 seconds). To minimize order effects, all items were randomized. Participants were allowed to skip any question they felt uncomfortable answering and received a thank-you message upon completion. To ensure data quality, responses that were incomplete, completed in under 30 seconds, or contained identical answers across all items were excluded. Data collection took place between May 7, 2024, and June 30, 2024. The ethical approval (approval number 202405001) was obtained from the Institutional Review Board of the School of Economics of Hangzhou Normal University. All participants provided oral informed consent. The components of oral informed consent were documented in writing and incorporated into the research proposal submitted to the Institutional Review Board, which subsequently granted ethical approval.

### 3.4 Data analysis

Using SPSS 25.0, Amos 24.0, and other software for data management and analysis, the primary analysis methods include reliability analysis, confirmatory factor analysis (CFA), descriptive statistics, correlation analysis, and structural equation model (SEM), and Bootstrap test (mediation effect test). Given that the measures for our core constructs utilized different Likert-scale formats (i.e., 5-point and 7-point scales), all variables were standardized prior to conducting the structural equation modeling and correlation analysis. This procedure ensures that the resulting coefficients are on a common scale and thus directly comparable.

### 3.5 Common method bias test

Given that all measures in this study were self-reported, there is a potential risk of common method bias (CMB). To assess this issue, we conducted Harman’s single-factor test following [80]. Additionally, we performed exploratory factor analysis (EFA) with principal component analysis (PCA) and confirmatory factor analysis (CFA), as recommended by Lindell [81]. The Kaiser-Meyer-Olkin (KMO) measure of sampling adequacy (0.945) and Bartlett’s test of sphericity (χ² = 53,924.768, df = 3,916, p < 0.001) confirmed that the data were appropriate for factor analysis [82]. The EFA results indicated that 18 factors with eigenvalues greater than 1 were extracted, with the largest single factor explaining only 21.964% of the total variance, well below the commonly accepted 40% threshold. These findings suggest that CMB is not a major concern in this study.

The KMO measure of sampling adequacy was 0.945, and Bartlett’s test of sphericity was significant (χ² = 53924.768, df = 3916, p < 0.001), confirming that the data were appropriate for factor analysis [83]. The EFA results indicated that 18 factors with eigenvalues greater than 1 were extracted, with the largest single factor explaining only 21.964% of the total variance, which is well below the commonly accepted 40% threshold. These findings suggest that CMB is not a major concern in this study. To further assess common method bias (CMB), a single-factor confirmatory factor analysis (CFA) was conducted. As shown in Table 2, the fit indices for the one-factor model were suboptimal. In contrast, the four-factor CFA model, which included emotional intelligence leadership (EIL), social support (SS), proactive personality (PP), and career decision-making self-efficacy (CDSE), demonstrated good model fit, meeting the criteria recommended by Hu and Bentler [75]. The comparison between the single-factor model and the four-factor model further supports that CMB is not a major issue in this study.

**Table 2:** Results of confirmatory factor analysis for common method bias assessment.

Modle	*χ²/df*	*GFI*	*AGFI*	*NFI*	*IFI*	*CFI*	*TLI*	*RMSEA*
Single-factor model (Harmon’s Test)	2.029	0.764	0.755	0.768	0.783	0.783	0.781	0.042
Four-factor model (Measurement Modle)	2.029	0.964	0.955	0.968	0.983	0.983	0.981	0.032

Note.The model fit threshold criteria are as follows: χ²/df < 3.00 (acceptable), CFI, TLI ≥ 0.95 (good fit), RMSEA ≤ 0.06 (good fit), and NFI, AGFI, IFI, GFI ≥ 0.90 (acceptable).

Nonetheless, given that self-reported measures were used, future research could benefit from employing multi-source data collection methods or longitudinal designs to further mitigate potential concerns regarding common method bias [80].

## 4. Results

### 4.1 Descriptive statistics and correlation analysis

Descriptive statistics and correlation analysis results are presented in Table 2. Emotional intelligence leadership, social support, proactive personality, and career decision-making self-efficacy were significantly and positively correlated. Emotional intelligence leadership was significantly positively correlated with social support (r = 0.251, p < 0.01), career decision-making self-efficacy (r = 0.487, p < 0.01), and proactive personality (r = 0.301, p < 0.01). Social support was significantly positively correlated with proactive personality (r = 0.243, p < 0.01) and career decision-making self-efficacy (r = 0.305, p < 0.01). These correlations provided support for subsequent hypothesis testing.

### 4.2 Mediation effect test

To account for the different measurement scales used, the structural equation model (SEM) was tested using standardized variables. In the structural equation model (SEM), emotional intelligence leadership served as the predictor, social support and proactive personality as mediators, and career decision-making self-efficacy as the outcome variable. The hypotheses were tested, and model fit was evaluated using AMOS 21.0. The results were as follows: χ²/df = 2.029, RMSEA = 0.032, IFI = 0.983, CFI = 0.983, TLI = 0.981. These indices demonstrate a high model fit, indicating that the mediation model is acceptable.

**Fig 2:**
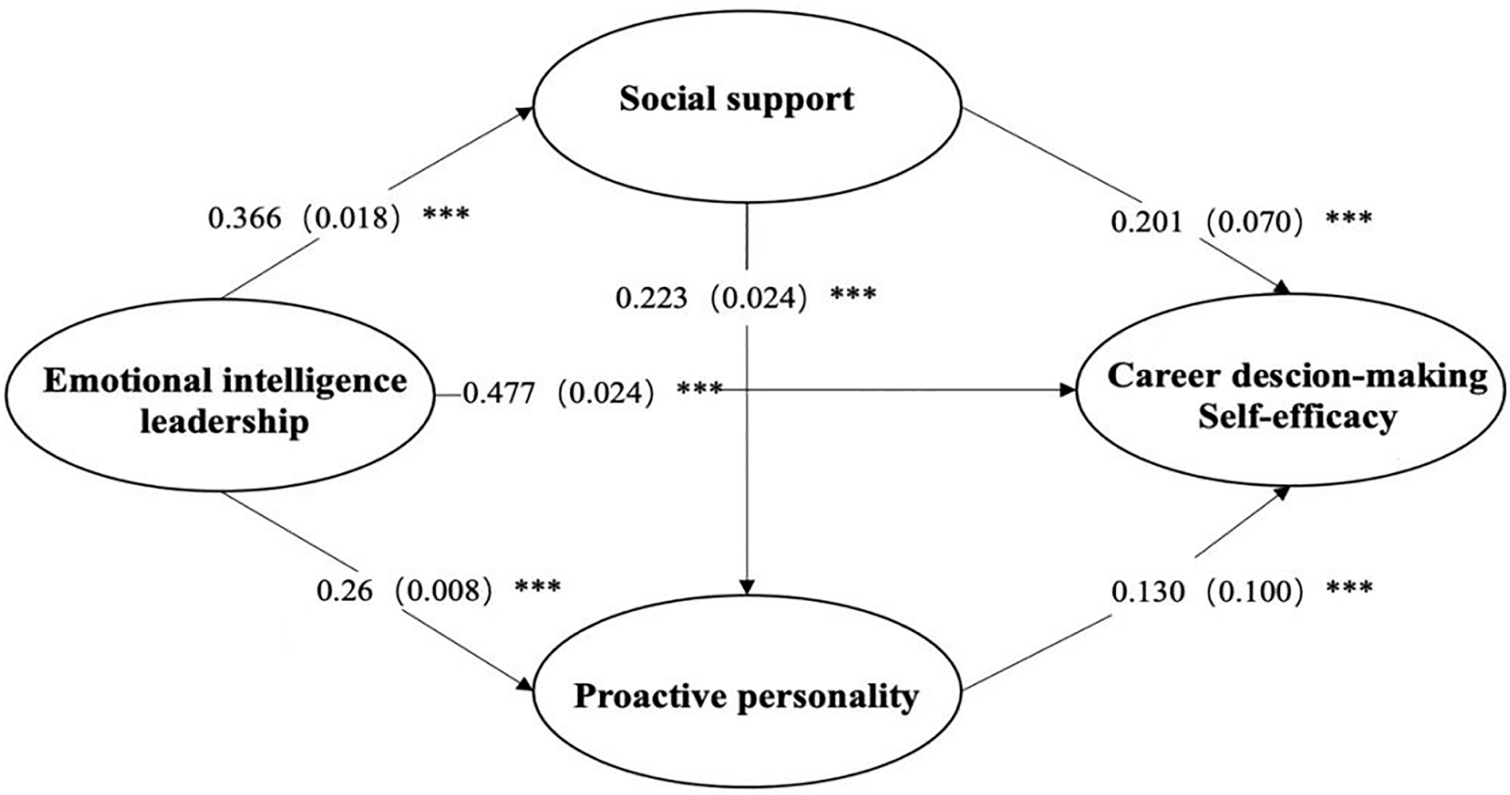
The final chain mediation model. (*p < 0.05,**p < 0.01,***p < 0.001. The model controls for gender, age, grade).

After controlling for variables such as gender and age, the mediation analysis (see Fig 2 and Table 3) revealed significant direct effects: emotional intelligence leadership was significantly positively associated with social support (β = 0.366, p < 0.001) and proactive personality (β = 0.260, p < 0.001). Emotional intelligence leadership also had a positive effect on career decision-making self-efficacy (β = 0.477, p < 0.001), while proactive personality was significantly positively associated with career decision-making self-efficacy (β = 0.130, p < 0.001). Additionally, social support had a significant positive effect on career decision-making self-efficacy (β = 0.201, p < 0.001) and was positively associated with proactive personality (β = 0.223, p < 0.001).

**Table 3:** Descriptive statistics and correlation analysis of all variables.

	*M ± SD*	*1*	*2*	*3*	*4*
1.Emotional Intelligence Leadership	101.270 *±* 19.104	1			
2.Social support	24.683 *±* 6.886	.251**	1		
3.Proactive personality	61.211 *±* 12.622	.301**	.243**	1	
4.Career decision-making self-efficacy	11.150 *±* 2.574	.487**	.305**	.331**	1

Note. N=996,*p < 0.05, **p < 0.01, and ***p < 0.001.

The mediation effect was assessed using the bias-corrected non-parametric Bootstrap method [83]. The 95% Bootstrap confidence interval excluded 0, indicating that social support and proactive personality significantly mediated the relationship between emotional intelligence leadership and career decision-making self-efficacy. As shown in Table 4, the total mediation effect was decomposed into three distinct indirect pathways:

**Table 4:** Structural Equation Model Fit Index.

	*χ²/df*	*CFI*	*NFI*	*AGFI*	*IFI*	*GFI*	*TLI*	*RMSEA*
Model	2.029	0.983	0.968	0.955	0.983	0.964	0.981	0.032

Note.The model fit threshold criteria are as follows: χ²/df < 3.00 (acceptable), CFI, TLI ≥ 0.95 (good fit), RMSEA ≤ 0.06 (good fit), and NFI, AGFI, IFI, GFI ≥ 0.90 (acceptable).

Path 1 (Mediation via Social Support): This pathway represents the indirect effect of emotional intelligence leadership on career decision-making self-efficacy through social support. The effect was significant (β = 0.074, 95% CI = [0.041, 0.114]), accounting for 12.77% of the total mediation effect.

Path 2 (Mediation via Proactive Personality): This pathway represents the indirect effect mediated specifically by proactive personality. The effect was also significant (β = 0.034, 95% CI = [0.013, 0.061]), accounting for 5.72% of the total mediation effect.

Path 3 (Chain Mediation): This pathway represents the serial indirect effect where emotional intelligence leadership influences social support, which in turn enhances proactive personality, ultimately affecting career decision-making self-efficacy. The chain mediation effect was significant (β = 0.011, 95% CI = [0.004, 0.022]), accounting for 1.85% of the total mediation effect.

The indirect effect of social support on career decision-making self-efficacy was significant (β = 0.074, 95% CI = 0.041 to 0.114, p < 0.001), accounting for 12.77% of the total mediation effect. Likewise, the indirect effect of proactive personality on career decision-making self-efficacy was significant (β = 0.034, 95% CI = 0.013 to 0.061, p < 0.01), accounting for 5.72% of the total mediation effect. Furthermore, the combined mediating effect of social support and proactive personality was significant (β = 0.011, 95% CI = 0.004 to 0.022, p < 0.01), accounting for 1.85% of the total mediation effect (see Tables 4 and 5).

**Table 5:** Chain mediation model.

Pathway	*β*	*SE*	*Boot* *LLCI*	*Boot* *ULCI*
Total effect	0.595	0.039	0.512	0.665
Direct effect	0.477	0.046	0.381	0.563
Indirect effect				
Emotional Intelligence Leadership →Social support →Career decision-making self-efficacy	0.074	0.018	0.041	0.114
Emotional Intelligence Leadership →Proactive personality →Career decision-making self-efficacy	0.034	0.012	0.013	0.061
Emotional Intelligence Leadership →Social support →Proactive personality→Career decision-making self-efficacy	0.011	0.004	0.004	0.022

Note.β = Standardized regression coefficient; SE = standard error; LCI = lower bound of 95% confidence interval; UCI = upper bound of 95% confidence interval.

## 5. Discussion

Grounded in SCCT, this study investigated the mechanisms linking emotional intelligence leadership with career decision-making self-efficacy among Chinese university students. Our findings not only confirm a significant direct positive relationship between these variables, consistent with prior research [26–28], but more importantly, reveal that social support and proactive personality form a critical serial mediation pathway. These results substantiate the core tenets of SCCT by demonstrating the dynamic interactions between person, environment, and behavior. Furthermore, our study extends SCCT by elucidating how these relationships operate within China’s collectivist cultural context, where familial and social support is particularly pivotal [84,85].

In line with H1, there is a positive correlation between emotional intelligence leadership and career decision-making self-efficacy. First, it enhances students’ emotional management and interpersonal skills, which are crucial for career development [3 5]. Second, it fosters a supportive environment that cultivates positive outcome expectations, thereby encouraging proactive career planning. Third, it improves relational networks, providing vital external resources for career exploration. Finally, it helps students recognize their self-worth, leading to more aligned career goal-setting [3 3,5 6].

The mediating roles of social support (H2) and proactive personality (H3) were also supported. Students with higher emotional intelligence leadership are more adept at building robust social support systems [86–88], which are crucial for navigating career challenges [45]. Similarly, emotional intelligence leadership fosters psychological safety and self-efficacy, encouraging proactive behaviors such as exploring opportunities and persevering through obstacles [56,57]. The mastery experiences derived from such behaviors subsequently strengthen career decision-making self-efficacy [2].

Confirming H4, the analysis supported the proposed serial mediation model, which represents the core theoretical contribution of this study. This effect illustrates a sequential process wherein environmental resources bolster personal traits, which in turn reinforce beliefs and behaviors. Specifically, emotional intelligence leadership is associated with greater access to social support. This support, often characterized in China by strong familial dependency [84], provides a secure base that reduces fear of failure and nurtures a proactive personality. Individuals who develop this proactive disposition are subsequently more likely to engage in active career planning, seek information, and persist through challenges, thereby accumulating positive experiences that enhance their career decision-making self-efficacy [89,90].

## 6. Implications and conclusion

### 6.1 Theoretical Implications

This study extends the Social Cognitive Career Theory (SCCT) framework [68] by introducing emotional intelligence leadership as a key personal factor. While prior research has indicated a relationship between emotional intelligence and career outcomes [26–28], this study empirically validates emotional intelligence leadership’s direct positive effect on career decision-making self-efficacy. This integration addresses a significant gap in extant literature regarding the specific role of emotional intelligence leadership in career decision-making contexts.

Second, it identifies a chain mediation pathway, showing how the positive association of emotional intelligence leadership with self-efficacy may pass through social support and then proactive personality. This finding helps clarify the dynamic interactions between personal traits, environmental factors, and behavioral outcomes as posited by the SCCT framework. This serial mediation model offers a comprehensive and nuanced explanation of the underlying mechanisms linking these variables.

Third, it tests and extends the SCCT framework within China’s collectivist culture, providing new evidence from a non-Western context. Consistent with studies emphasizing the influence of cultural context on career development [19,20,91], our findings advance SCCT by offering a novel cross-cultural perspective. This evidences the critical role of culture in shaping career development mechanisms and enriches the theory’s applicability across diverse cultural contexts.

### 6.2 Practical implications

The findings of this study offer actionable recommendations for higher education institutions, student support services, and students, proposing an integrated approach to bolster career decision-making self-efficacy.

For higher education institutions, the results advocate for a shift from isolated workshops to a holistic integration of emotional intelligence leadership development across academic and co-curricular programs [92,93]. Competencies such as empathy and self-regulation should be embedded into curricula, particularly within team-based projects and student organization management. This approach ensures students actively practice leadership, which is a critical first step toward building the robust social support networks essential for career development. By cultivating emotional intelligence leadership, universities empower students to build and leverage their own support systems [94].

For student support services, this study illuminates a more strategic intervention pathway. Rather than providing fragmented resources, these departments should act as systemic facilitators of this developmental process [95]. Initiatives should first help students leverage their emotional intelligence to build supportive relationships. Subsequently, departments can guide students to use this support system as a secure “scaffolding” to foster proactive behaviors, such as seeking informational interviews or undertaking internships. This reframes the role of student support from merely providing comfort to actively cultivating a proactive mindset [96–98].

Within the Chinese cultural context, where familial influence is significant, institutions should develop targeted programs involving parents, such as joint career planning workshops. Furthermore, emotional intelligence leadership training can coach students on how to effectively communicate career aspirations to their families and manage differing expectations, turning a potential source of pressure into a pillar of support.

### 6.3 Conclusion

Grounded in Social Cognitive Career Theory (SCCT), this study constructed an integrated model to explore how emotional intelligence leadership influences career decision-making self-efficacy among Chinese university students. The empirical results yield three key conclusions.

First, emotional intelligence leadership serves as a significant positive predictor of career decision-making self-efficacy, highlighting its role as an intrinsic driver for career confidence. Second, the study confirms the mediating roles of social support and proactive personality, indicating that emotional intelligence leadership enhances self-efficacy by fostering supportive networks and activating individual agency. Third, and most critically, a serial mediation mechanism was validated. This reveals a progressive pathway wherein emotional intelligence leadership facilitates the acquisition of social support, which in turn nurtures a proactive personality, ultimately leading to robust career decision-making self-efficacy.

In summary, this research not only empirically supports the value of emotional intelligence leadership in career development but also clarifies the complex “person-environment” mechanisms underlying this relationship. These findings suggest that systematically cultivating leadership competencies and supportive environments provides a viable pathway to enhance college students’ informed career decision-making..

## 7. Limitations and future research

Despite the theoretical and practical contributions, this study has several limitations that should be acknowledged and addressed in future research.

First, the cross-sectional design limits our ability to make strict causal inferences among the variables. While the structural equation modeling provides support for the hypothesized pathways, the directionality of relationships is theoretical. Future research should adopt a longitudinal design to continuously track the developmental trajectories of emotional intelligence leadership, social support, proactive personality, and career decision-making self-efficacy in the same group of students. This approach would verify causal relationships and reveal dynamic changes during the career development process, providing an empirical basis for precise interventions at specific educational stages [99].

Second, the sample was drawn exclusively from university students in China, which limits the generalizability of the findings to other cultural contexts. Given that career decision-making is deeply influenced by cultural values (e.g., collectivism vs. individualism), future research should expand the sample to different cultural and geographical contexts. Comparative studies could examine whether the mechanisms of emotional intelligence leadership differ across cultures, enhancing our understanding of how culture shapes leadership development and student career decisions [19,20].

Third, the data were obtained entirely through self-report measures. Although statistical tests indicated that common method bias was not a major concern [80], self-reported data may still be subject to social desirability bias. To address this, future research could employ multi-source data collection (e.g., incorporating peer or teacher ratings of leadership) or experimental designs. Specifically, developing and implementing targeted intervention programs(e.g., workshops aiming at enhancing emotional intelligence leadership)would allow researchers to evaluate actual effects on career decision-making self-efficacy through experimental and control group comparisons [100].

## Supporting information

S1 File Survey Questionnaire. This file contains the full set of survey items used in the study.
(DOCX)

S2 File Raw data of the study. This file includes the anonymized data collected from university students.
(XLSX)
