# Supplementary material for: Emotional intelligence leadership and career decision-making self-efficacy among college students in China: The mediating role of social support and proactive personality
Source: PLoS One. 2026 Feb 23;21(2):e0343432. doi: 10.1371/journal.pone.0343432 (PMC12928404; doi:10.1371/journal.pone.0343432)
Supplement: S1 File — This file contains the full set of survey items used in the study. (DOCX) [file pone.0343432.s001.docx]

**Survey Questionnaire on College Student Leadership Education**

Hello! Thank you for taking the time to participate in our Survey on College Student Leadership. Please read the following statements carefully and respond according to your actual situation. Your participation is greatly appreciated! This survey is anonymous, and the results will be used for scientific research purposes only. The process of completing this questionnaire is also a learning opportunity for leadership; we look forward to you becoming an outstanding leader!

1.Gender:

□ Male □ Female

2.Your age is: (Fill in the blank)

3.Current Grade:

□ Freshman □ Sophomore □ Junior □ Senior □ 5th Year Undergraduate

4.Your Discipline:

□ Philosophy □ Economics □ Law □ Education □ Literature □ History □ Science □ Engineering □ Agriculture □ Medicine □ Military Science □ Management □ Arts

5.Which province are you from: (Fill in the blank)

6.Your comprehensive academic ranking in your class, major, or grade:

□ 0-15% □ 15%-30% □ 30%-50% □ 50%-70% □ 70%-100%

1. How many close friends do you have who can provide support and help?

□ None □ 1-2 □ 3-5 □ More than 6

8.In the past year, you:

□ Lived away from family and lived alone □ Moved frequently, mostly living with strangers □ Lived with classmates or friends □ Lived with family members

9.Relationship with roommates:

□ No mutual concern, just acquaintances □ Occasional concern when encountering difficulties □ Some roommates care about you □ Most roommates care about me

10.Relationship with classmates:

□ No mutual concern, just acquaintances □ Occasional concern when encountering difficulties □ Some classmates care about you □ Most classmates care about me

11.Support and care received from family members or school:

□ Parents a. None b. Very Little c. Average d. Full Support □ Partner a. None b. Very Little c. Average d. Full Support □ Siblings a. None b. Very Little c. Average d. Full Support □ Other members a. None b. Very Little c. Average d. Full Support

12.In the past, sources of financial support and practical help during crises (Multiple Choice):

□ No source □ Following sources: a. Partner b. Family c. Relatives d. Classmates e. Friends f. Subject Teacher g. Counselor h. Party/Youth League Leaders/Teachers i. Online Friends j. Other organizations/individuals (please list): ______

13.In the past, sources of comfort and care during crises (Multiple Choice):

□ No source □ Following sources: a. Partner b. Family c. Relatives d. Classmates e. Friends f. Subject Teacher g. Counselor h. Party/Youth League Leaders/Teachers i. Online Friends j. Other organizations/individuals (please list): ______

14.How you confide in others when in trouble:

□ Never tell anyone □ Only tell 1-2 very close people □ Tell if friends ask □ Actively share troubles to obtain support and understanding

15.How you seek help when troubled:

□ Rely only on myself, accept no help □ Rarely ask others for help □ Sometimes ask for help □ Frequently seek help from family, friends, or organizations

16.For activities organized by groups, such as (Party/Youth League organizations, Student Unions, etc.):

□ Never participate in any; □ Occasionally participate; □ Frequently participate □ Actively participate and lead activities

1. Number of times you have taken leadership-related elective courses (e.g., Writing and Communication, Career Development, Entrepreneurship Education, etc.) during school:

□ 0 times (Never) □ 1-2 times (Occasionally) □ 3-4 times (Sometimes) □ 5-6 times (Frequently) □ More than 6 times (Always)

18.Number of times you have participated in specialized college/departmental leadership programs during school:

□ 0 times (Never) □ 1-2 times (Occasionally) □ 3-4 times (Sometimes) □ 5-6 times (Frequently) □ More than 6 times (Always)

19.Number of times you have participated in student organizations during school:

□ 0 times (Never) □ 1-2 times (Occasionally) □ 3-4 times (Sometimes) □ 5-6 times (Frequently) □ More than 6 times (Always)

20.Number of times you have served as a leader in student organizations during school:

□ 0 times (Never) □ 1-2 times (Occasionally) □ 3-4 times (Sometimes) □ 5-6 times (Frequently) □ More than 6 times (Always)

21.Number of times you have participated in social practice during school:

□ 0 times (Never) □ 1-2 times (Occasionally) □ 3-4 times (Sometimes) □ 5-6 times (Frequently) □ More than 6 times (Always)

22.Number of times you have participated in volunteer services during school:

□ 0 times (Never) □ 1-2 times (Occasionally) □ 3-4 times (Sometimes) □ 5-6 times (Frequently) □ More than 6 times (Always)

23.Number of times you have engaged in part-time social jobs during school:

□ 0 times (Never) □ 1-2 times (Occasionally) □ 3-4 times (Sometimes) □ 5-6 times (Frequently) □ More than 6 times (Always)

24.Number of times you have participated in Red/Patriotic Education (e.g., reading Red classics, visiting Red bases, watching Red movies) during school:

□ 0 times (Never) □ 1-2 times (Occasionally) □ 3-4 times (Sometimes) □ 5-6 times (Frequently) □ More than 6 times (Always)

25.Spend time understanding informal traditions (unwritten rules) in the team:

□ Never □ Rarely □ Sometimes □ Frequently □ Always

26.Understand the explicit and latent values of the team:

□ Never □ Rarely □ Sometimes □ Frequently □ Always

27.Pay attention to how my emotions affect my collaboration with others:

□ Never □ Rarely □ Sometimes □ Frequently □ Always

28Work as hard as possible:

□ Never □ Rarely □ Sometimes □ Frequently □ Always

1. Adjust my leadership style to fit the situation:

□ Never □ Rarely □ Sometimes □ Frequently □ Always

1. Utilize my strengths:

□ Never □ Rarely □ Sometimes □ Frequently □ Always

1. Understand how the team environment affects my leadership style:

□ Never □ Rarely □ Sometimes □ Frequently □ Always

1. Help others improve their skills and abilities

□ Never □ Rarely □ Sometimes □ Frequently □ Always

1. Understand the interrelationships among team members:

□ Never □ Rarely □ Sometimes □ Frequently □ Always

1. Keep promises:

□ Never □ Rarely □ Sometimes □ Frequently □ Always

1. Build a sense of tea:

□ Never □ Rarely □ Sometimes □ Frequently □ Always

1. Reflect on how my leadership style fits the team culture:

□ Never □ Rarely □ Sometimes □ Frequently □ Always

1. Create a positive and optimistic atmosphere:

□ Never □ Rarely □ Sometimes □ Frequently □ Always

1. Consider how my decisions are accepted by team member:

□ Never □ Rarely □ Sometimes □ Frequently □ Always

1. Improve my abilities:

□ Never □ Rarely □ Sometimes □ Frequently □ Always

1. Identify external factors that impact the team:

□ Never □ Rarely □ Sometimes □ Frequently □ Always

1. Consider the needs of other team members:

□ Never □ Rarely □ Sometimes □ Frequently □ Always

1. Discern various behavioral patterns within the team:

□ Never □ Rarely □ Sometimes □ Frequently □ Always

1. Reflect on how my actions align with my own values:

□ Never □ Rarely □ Sometimes □ Frequently □ Always

1. Understand the strengths of team members:

□ Never □ Rarely □ Sometimes □ Frequently □ Always

1. Listen carefully to what team members are saying:

□ Never □ Rarely □ Sometimes □ Frequently □ Always

1. Work with team members toward a common goal:

□ Never □ Rarely □ Sometimes □ Frequently □ Always

47.Work to resolve conflicts within the team:

□ Never □ Rarely □ Sometimes □ Frequently □ Always

48.Coordinate differing viewpoints within the team:

□ Never □ Rarely □ Sometimes □ Frequently □ Always

49.For what I expect others to do, I set an example:

□ Never □ Rarely □ Sometimes □ Frequently □ Always

50.I look forward to the future and communicate with others about the things I see that may affect us in the future:

□ Never □ Rarely □ Sometimes □ Frequently □ Always

1. I look for various ways to develop and challenge my potential:

□ Never □ Rarely □ Sometimes □ Frequently □ Always

1. In the work process, I prefer to foster cooperation rather than competitive relationships:

□ Never □ Rarely □ Sometimes □ Frequently □ Always

1. I praise people who complete work tasks excellently:

□ Never □ Rarely □ Sometimes □ Frequently □ Always

1. I spend time and energy ensuring that team members follow principles and norms that everyone has agreed upon:

□ Never □ Rarely □ Sometimes □ Frequently □ Always

1. I articulate the goals we should be capable of achieving to team members:

□ Never □ Rarely □ Sometimes □ Frequently □ Always

1. I work to seek various methods to encourage everyone to innovate:

□ Never □ Rarely □ Sometimes □ Frequently □ Always

1. I actively listen to various different opinions:

□ Never □ Rarely □ Sometimes □ Frequently □ Always

1. I encourage members in the team.

□ Never □ Rarely □ Sometimes □ Frequently □ Always

1. I keep promises within the organization:

□ Never □ Rarely □ Sometimes □ Frequently □ Always

60.I share the vision for the positive development of the organization with organization members:□ Never □ Rarely □ Sometimes □ Frequently □ Always

61.I stay attentive to events and activities that may affect the organization:

□ Never □ Rarely □ Sometimes □ Frequently □ Always

1. I respect others:

□ Never □ Rarely □ Sometimes □ Frequently □ Always

1. I not only provide support for members in the organization but also express affirmation and appreciation for their contributions:

□ Never □ Rarely □ Sometimes □ Frequently □ Always

1. Regarding how my actions affect the performance of others, I seek various ways to get feedback:

□ Never □ Rarely □ Sometimes □ Frequently □ Always

1. I discuss with others how to achieve a win-win situation by working towards common goals:

□ Never □ Rarely □ Sometimes □ Frequently □ Always

66.When things develop unexpectedly, I ask myself: What can we learn from these experiences? □ Never □ Rarely □ Sometimes □ Frequently □ Always

67.I support other members in the organization to make independent decisions:

□ Never □ Rarely □ Sometimes □ Frequently □ Always

1. For those who follow our values, I take special care to publicly acknowledge them:

□ Never □ Rarely □ Sometimes □ Frequently □ Always

1. I establish organizational values based on team consensus:

□ Never □ Rarely □ Sometimes □ Frequently □ Always

70.When discussing the goals that our organization strives for, I am always optimistic and positive: □ Never □ Rarely □ Sometimes □ Frequently □ Always

71.For the projects to be undertaken, I ensure that we have set goals and formulated detailed plans: □ Never □ Rarely □ Sometimes □ Frequently □ Always

72.I give others a lot of freedom and choice to let them decide how to carry out their work:

□ Never □ Rarely □ Sometimes □ Frequently □ Always

1. I seek various ways for everyone to celebrate success together:

□ Never □ Rarely □ Sometimes □ Frequently □ Always

1. I talk with others about the values and principles that guide my success:

□ Never □ Rarely □ Sometimes □ Frequently □ Always

1. I believe what we do is for a higher goal, very meaningful and valuable; whenever talking about this, I am very firm:

□ Never □ Rarely □ Sometimes □ Frequently □ Always

1. I am courageous to try working in different ways within the organization:

□ Never □ Rarely □ Sometimes □ Frequently □ Always

1. I provide others with opportunities to take on leadership responsibilities:

□ Never □ Rarely □ Sometimes □ Frequently □ Always

1. I am certain that in our organization, everyone can be creatively recognized for their contributions:

□ Never □ Rarely □ Sometimes □ Frequently □ Always

1. If I see others in difficulty, I will do my best to provide help:

□ Strongly Disagree □ Somewhat Disagree □ Disagree □ Uncertain □ Agree □ Somewhat Agree □ Strongly Agree

1. I am good at turning problems into opportunities:

□ Strongly Disagree □ Somewhat Disagree □ Disagree □ Uncertain □ Agree □ Somewhat Agree □ Strongly Agree

1. I am always looking for better ways to do things:

□ Strongly Disagree □ Somewhat Disagree □ Disagree □ Uncertain □ Agree □ Somewhat Agree □ Strongly Agree

1. When encountering problems, I face them directly:

□ Strongly Disagree □ Somewhat Disagree □ Disagree □ Uncertain □ Agree □ Somewhat Agree □ Strongly Agree

1. I like to challenge the status quo:

□ Strongly Disagree □ Somewhat Disagree □ Disagree □ Uncertain □ Agree □ Somewhat Agree □ Strongly Agree

1. If I believe in an idea, no obstacle can stop me from achieving it:

□ Strongly Disagree □ Somewhat Disagree □ Disagree □ Uncertain □ Agree □ Somewhat Agree □ Strongly Agree

85.If I firmly believe in something, regardless of the possibility of success or failure, I will do it: □ Strongly Disagree □ Somewhat Disagree □ Disagree □ Uncertain □ Agree □ Somewhat Agree □ Strongly Agree

86.Nothing is more exciting than seeing my ideas become reality:

□ Strongly Disagree □ Somewhat Disagree □ Disagree □ Uncertain □ Agree □ Somewhat Agree □ Strongly Agree

1. I am always looking for new ways to make my life better:

□ Strongly Disagree □ Somewhat Disagree □ Disagree □ Uncertain □ Agree □ Somewhat Agree □ Strongly Agree

1. I enjoy the pleasure brought by facing and overcoming obstacles in ideas:

□ Strongly Disagree □ Somewhat Disagree □ Disagree □ Uncertain □ Agree □ Somewhat Agree □ Strongly Agree

1. I always hope that I am special in the group (perhaps in this world):

□ Strongly Disagree □ Somewhat Disagree □ Disagree □ Uncertain □ Agree □ Somewhat Agree □ Strongly Agree

1. Able to accurately assess my own abilities:

□ No confidence at all □ Very little confidence □ Moderate confidence □ Much confidence □ Complete confidence

1. Able to find information about careers of interest:

□ No confidence at all □ Very little confidence □ Moderate confidence □ Much confidence □ Complete confidence

1. Able to choose a major from the candidate major list considered:

□ No confidence at all □ Very little confidence □ Moderate confidence □ Much confidence □ Complete confidence

1. Able to formulate a five-year goal plan:

□ No confidence at all □ Very little confidence □ Moderate confidence □ Much confidence □ Complete confidence

1. If encountering learning difficulties in the chosen major, able to make decisions on the measures to be taken:

□ No confidence at all □ Very little confidence □ Moderate confidence □ Much confidence □ Complete confidence

1. Able to determine what my ideal job will be:

□ No confidence at all □ Very little confidence □ Moderate confidence □ Much confidence □ Complete confidence

1. Able to find out the employment trend of a career in the next decade:

□ No confidence at all □ Very little confidence □ Moderate confidence □ Much confidence □ Complete confidence

1. Able to choose a career from the candidate career list considered:

□ No confidence at all □ Very little confidence □ Moderate confidence □ Much confidence □ Complete confidence

1. Able to make decisions on the measures needed to successfully complete the chosen major:

□ No confidence at all □ Very little confidence □ Moderate confidence □ Much confidence □ Complete confidence

1. Even when encountering setbacks, still able to persist in dedicating to one's major or career goals:

□ No confidence at all □ Very little confidence □ Moderate confidence □ Much confidence □ Complete confidence

1. Even when encountering setbacks, still able to persist in dedicating to one's major or career goals:

□ No confidence at all □ Very little confidence □ Moderate confidence □ Much confidence □ Complete confidence

1. Able to clarify what things I consider most valuable in a profession:

□ No confidence at all □ Very little confidence □ Moderate confidence □ Much confidence □ Complete confidence

102.Able to find out the average annual income of a profession:

□ No confidence at all □ Very little confidence □ Moderate confidence □ Much confidence □ Complete confidence

103.Able to choose a career that suits my favorite lifestyle:

□ No confidence at all □ Very little confidence □ Moderate confidence □ Much confidence □ Complete confidence

104.Able to prepare a good resume:

□ No confidence at all □ Very little confidence □ Moderate confidence □ Much confidence □ Complete confidence

105.If I don't like the major chosen for the first time, I am able to change majors:

□ No confidence at all □ Very little confidence □ Moderate confidence □ Much confidence □ Complete confidence

106.Able to clarify what I plan to sacrifice and not sacrifice in order to achieve my career goals: □ No confidence at all □ Very little confidence □ Moderate confidence □ Much confidence □ Complete confidence

107.Able to talk to a person who is already working in the career field you are interested in:

□ No confidence at all □ Very little confidence □ Moderate confidence □ Much confidence □ Complete confidence

108.Able to stop worrying about whether the decision was correct after making a career decision: □ No confidence at all □ Very little confidence □ Moderate confidence □ Much confidence □ Complete confidence

109.Able to identify people, companies, and research institutes related to my future career:

□ No confidence at all □ Very little confidence □ Moderate confidence □ Much confidence □ Complete confidence

110.If not satisfied with the career engaged in, able to change jobs:

□ No confidence at all □ Very little confidence □ Moderate confidence □ Much confidence □ Complete confidence

111.Able to clearly describe what the lifestyle I want is:

□ No confidence at all □ Very little confidence □ Moderate confidence □ Much confidence □ Complete confidence

12.Able to find information about graduation institutions:

□ No confidence at all □ Very little confidence □ Moderate confidence □ Much confidence □ Complete confidence

113.Able to choose a major or career that fits my interests:

□ No confidence at all □ Very little confidence □ Moderate confidence □ Much confidence □ Complete confidence

1. Able to successfully handle the job interview process:

□ No confidence at all □ Very little confidence □ Moderate confidence □ Much confidence □ Complete confidence

1. If I cannot get the first choice major or career, I am able to accept some other reasonable majors or careers as substitutes:

□ No confidence at all □ Very little confidence □ Moderate confidence □ Much confidence □ Complete confidence
